# Extracellular host DNA contributes to pathogenic biofilm formation during periodontitis

**DOI:** 10.3389/fcimb.2024.1374817

**Published:** 2024-05-08

**Authors:** Mariana Slobodianyk-Kolomoiets, Svitlana Khlebas, Iryna Mazur, Kateryna Rudnieva, Viktoria Potochilova, Olga Iungin, Olexandr Kamyshnyi, Iryna Kamyshna, Geert Potters, Andrew J. Spiers, Olena Moshynets

**Affiliations:** ^1^ Department of Dentistry, Shupyk National Healthcare University of Ukraine, Kyiv, Ukraine; ^2^ Central Clinical Diagnostic Laboratory, Kyiv Regional Clinical Hospital, Kyiv, Ukraine; ^3^ Department of Microbiology, Virology and Immunology, Bogomolets National Medical Academy, Kyiv, Ukraine; ^4^ Bacteriological laboratory, Kyiv City Maternity Hospital № 2, Kyiv, Ukraine; ^5^ Biofilm Study Group, Department of Cell Regulatory Mechanisms, Institute of Molecular Biology and Genetics, National Academy of Sciences of Ukraine, Kyiv, Ukraine; ^6^ Department of Biotechnology, Leather and Fur, Faculty of Chemical and Biopharmaceutical Technologies, Kyiv National University of Technologies and Design, Kyiv, Ukraine; ^7^ Microbiology, Virology and Immunology Department, I. Horbachevsky Ternopil National Medical University, Ternopil, Ukraine; ^8^ Antwerp Maritime Academy, Antwerp, Belgium; ^9^ Department of Bioscience Engineering, University of Antwerp, Antwerp, Belgium; ^10^ School of Applied Sciences, Abertay University, Dundee, United Kingdom

**Keywords:** periodontitis, subgingival biofilms, eDNA, biofilm structure, FISH

## Abstract

**Introduction:**

Periodontal diseases are known to be associated with polymicrobial biofilms and inflammasome activation. A deeper understanding of the subgingival cytological (micro) landscape, the role of extracellular DNA (eDNA) during periodontitis, and contribution of the host immune eDNA to inflammasome persistence, may improve our understanding of the mechanisms underlaying severe forms of periodontitis.

**Methods:**

In this work, subgingival biolfilms developing on biologically neutral polyethylene terephthalate films placed in gingival cavities of patients with chronic periodontitis were investigated by confocal laser scanning microscopy (CLSM). This allowed examination of realistic cytological landscapes and visualization of extracellular polymeric substances (EPS) including amyloids, total proteins, carbohydrates and eDNA, as well as comparison with several single-strain *in vitro* model biofilms produced by oral pathogens such as *Klebsiella pneumoniae*, *Pseudomonas aeruginosa*, *Staphylococcus aureus*, *Streptococcus gordonii*, *S. sanguinis* and *S. mitis*. Fluorescence *in situ* hybridization (FISH) analysis was also used to identify eDNA derived from eubacteria, streptococci and members of the *Bacteroides–Porphyromonas–Prevotella* (BPP) group associated with periodontitis.

**Results:**

Analysis of subgingival biofilm EPS revealed low levels of amyloids and high levels of eDNA which appears to be the main matrix component. However, bacterial eDNA contributed less than a third of the total eDNA observed, suggesting that host-derived eDNA released in neutrophil extracellular traps may be of more importance in the development of biofilms causing periodontitis.

**Discussion:**

eDNA derived from host immunocompetent cells activated at the onset of periodontitis may therefore be a major driver of bacterial persistence and pathogenesis.

## Introduction

Biofilm formation is a bacterial strategy allowing the colonization of an ecological niche (econiche) (Moshynets and Spiers, 2016). Biofilms are three-dimensional structures where sessile cells reside and are protected by self-produced matrix of extracellular polymeric substances (EPS). These structures make bacteria less susceptible to different environmental stressors and help them to communicate with each other, to coordinate their metabolism, and to better adapt to the changing environment (McLaughlin et al., 2017; Moshynets et al., 2018). Moreover, biofilms are central to a wide range of infection processes, as any bacterial infection eventually involves biofilm formation which is further confounded by interactions with the host immune response.

Periodontal diseases have been recognized as the result of infection and inflammation of the gums and bone that surround and support the teeth (CDC, July 10, 2013; www.cdc.gov/oralhealth/conditions/periodontal-disease, accessed on 13 November 2023). They are considered to occur in close association with aggressive bacterial colonization and pathogenic biofilm formation, and include *Escherichia, Enterococcus, Klebsiella, Lactococcus, Pseudomonas, Staphylococcus* and *Streptococcus* species (Ansiliero et al., 2021; Emery et al., 2021; Espíndola et al., 2022), plus the more pathogenic, secondary-colonizing *Aggregatibacter actinomycetemcomitans*, *Fusobacterium nucleatum*, *Porphyromonas gingivalis*, *Tannerella forsythia*, and *Treponema denticola* species (Kuboniwa and Lamont, 2010; Zijnge et al., 2010; Könönen et al., 2019; Diaz and Valm, 2020) in diverse communities where spatial interactions are important in supragingival biofilms as well as in plagues formed above the gum (Mark Welch et al., 2016; Zijnge et al., 2010).

While the generally acknowledged process explaining the onset and development of periodontitis considers the role of pathogenic biofilm persistence, there are a number of questions that remain unanswered, and our understanding of how pathogenic multispecies biofilms interact with the host immune system is incomplete. Firstly, it is not yet understood why the bacterial community in the dental pocket of healthy people and periodontitis patients are essentially the same (van Winkelhoff et al., 2002; Casado et al., 2011; Naginyte et al., 2019). Secondly, subgingival biofilms are also present in healthy individuals and are in a balanced relationship with the host and it is only when this equilibrium is altered that the inflammasome is activated which leads to periodontitis manifestation (Ding et al., 2020; Esparbès et al., 2021). There is also a measure of heritability with up to a third of the variance of periodontitis due to genetic factors, with higher heritability for more severe disease (Nibali et al., 2019). Finally, little is understood about how the spatial structure of subgingival and supragingival biofilms might differ despite developing in significantly different econiches. In supragingival biofilms, the dental plaque community was shown to consist of a radial nine-taxon structure organized around filamentous corynebacteria with individual taxa spatially distributed at the micrometer scale according to their functional niche (Mark Welch et al., 2016). As well as bacteria, yeast such as Candida have also been found in oral biofilms and associated with periodontal disease (Jakubovics et al., 2021). The matrix of supragingival biofilms is also complex with a range of EPS including secreted and surface-associated amyloid fibers, other proteins and polysaccharides, as well as cellular debris, and bacterial extracellular DNA (eDNA) (Tawakoli et al., 2017; Jakubovics et al., 2021). However, a better spatial understanding of how these components might interact in oral and subgingival biofilms is required, and what role host eDNA released due to neutrophil extracellular traps (NET) activation (NETosis) and autoimmune reaction (Palaniyar et al., 2005; Oveisi et al., 2019) may have in biofilm formation and persistence.

Here we present an adaptation of the modified Cholodny method using biologically neutral polyethylene terephthalate (PET) films to sample real cytological (micro) landscapes which develop during active periodontitis (Moshynets et al., 2011). This method allows for a spatial analysis of both matrix components and cell-cell interactions in subgingival biofilms, and points towards a number of biological conditions guiding the development of periodontitis.

## Materials and methods

### Ethics statement

Ethics approval was granted by the Ethics Committee of from the Shupyk National Healthcare University of Ukraine (protocol No 8 dated by 7 November 2022) and written informed consent obtained from all participants before inclusion in the study.

### Sample collection

All patients were identified as having chronic periodontitis with no systemic disorders after clinical examination which included analysis of the plaque-control record, recording the probing depth and attachment level at six sites around each tooth, and assessment of alveolar bone resorption. No patient had a history of periodontal treatment and none had taken antibiotics in the three months prior to the baseline examination. Sampling of subgingival cytological landscapes was performed by allowing natural biofouling of biologically neutral polyethylene terephthalate (PET) films (2 – 4 x 7 mm) placed in periodontal spaces for five days. Briefly, each film was inserted in a gingival cavity where periodontitis of patients having moderate to advanced periodontitis stage was diagnosed. The patients were instructed to follow their normal oral hygiene but were requested to take care when brushing near the tooth with the film. After five days, the films were removed and placed in a plastic micro-tube and immediately delivered for cytological analysis. When needed, 1 mg/ml DNase (Sigma-Aldrich), dissolved in a buffer containing 20 mM Tris-HCl (pH 7.4) and 10 mM MgCl_2_ (Sambrook et al., 1989), was applied for 20 min before staining the samples and subsequent microscopy imaging.

### Bacteria and culturing conditions

*Escherichia coli* K12, *Klebsiella penumoniae* ATCC 10031, *Pseudomonas aeruginosa* PA01, *P. fluorescens* SBW25, and *Staphylococcus aureus* ATCC 29423, were obtained from laboratory stock collections (Moshynets et al., 2020; Chernii et al., 2021; Moshynets et al., 2022b; Volynets et al., 2022). The Ukrainian hospital isolates *K. pneumoniae* UHI 489 and UHI 117 were from previous work (Moshynets et al., 2022a) and three oral streptococcal opportunistic pathogenic isolates, *Streptococcus gordonii* UHI1, *S. sanguinis* UHI1 and *S. mitis* UHI1, were isolated from samples collected from patients of the Maxillofacial Surgery Department of the Kyiv Regional clinical hospital with purulent-inflammatory processes and identified using a Bacteriological Analyzer Vitek 2 Compact v9 (bioMérieux, France). Bacteria were incubated aerobically at 37°C except for *P. fluorescens* SBW25 which was cultured at 28°C. Over-night shaken Luria-Bertani (LB) liquid cultures provided 300 µl inocula for biofilm assays which were undertaken in static 30 ml microcosms containing 5 ml media. These were incubated at 28°C or 37°C and biofilms harvested after three days. Solid-liquid interface biofilms formed on the bottom surface of microcosms, except for *Pf* SBW25 where air-liquid interface biofilms were formed (Moshynets et al., 2022c).

### Microscopic analysis

Biofilm and cytological analysis was performed using confocal laser scanning microscopy (CLSM). Biofilm samples obtained from microcosms, cytological samples obtained with a spoon excavator, and subgingival biofilms formed on biologically neutral polyethylene terephthalate (PET) films, were transferred to microscopic glass slides prior to staining. Fluorescent stains were used to visualize intracellular DNA (iDNA) and extracellular DNA (eDNA), amyloids, total protein, and Candida cells. Stain combinations were based on complementary excitation/emission (Ex/Em) wavelengths which allowed individual stains to be visualized using separate CLSM channels.

iDNA was visualized with 5 µM SYBR Green solution (Thermo Fisher Scientific) which can be used to stain live cells with an intact membrane or dead cells or those with a damaged membrane and was used as a proxy for bacterial cells. 2 µg/ml Ethidium bromide solution (Sigma-Aldrich, UK) was also used to visualize iDNA because under the conditions we used, the eDNA signal was found to be very diffused compared to the signals coming from intact cells. eDNA was visualised with 20 µg/ml Propidium iodide in water, or 1 mM of the experimental stain 986 (Moshynets et al., 2022c) (supplied by SM Yarmoluk) in DMSO, which do not penetrate intact cells. Double-staining eDNA with Propidium iodide and SYBR Green is not possible as this dye combination results in fluorescence resonance energy transfer (FRET) reducing SYBR Green fluorescence and increasing Propidium iodide fluorescence (Barbesti et al., 2000). Amyloids were visualised with 1 mM AmyGreen solution [of our own synthesis (Moshynets et al., 2020)] in DMSO and total proteins with 0.5 µg/ml Thiazine Red R (Sigma) in 0.1% acetic acid. Carbohydrates (β(1-3) glucans) and Candida cells (β(1-4) glucan, i.e., chitin) were visualised with 2 µg/ml Calcofluor White (Sigma-Aldrich, UK) in water (Domenech et al., 2013; Rezende et al., 2019).

No additional washing after staining was performed in order to limit the physical disruption of samples during preparation, and a cover slip was placed over the samples before imaging. CLSM analysis was undertaken using a Leica TCS SPE Confocal system with coded DMi8 inverted microscope (Leica, Germany) and Leica Application Suite X (LAS X) v3.4.1. Representative images were acquired using a maximum of three channels for SYBR Green or AmyGreen (Ex/Em 488/490 – 580 nm), Ethidium bromide, 986, Propidium iodide or Thiazine Red R (532/537 – 670 nm), and Calcofluor White (405/450 – 500 nm), with total pixel numbers determined for each stain (channel) using LAS X. Data are shown as total abundance or as the relative abundance compared to iDNA levels.

### Fluorescence *in situ* hybridization

Analysis of biofilm samples by FISH followed earlier work (Hannig et al., 2007). Oligonucleotide probes for eubacteria, streptococci and the *Bacteroides–Porphyromonas–Prevotella* (BPP) group were synthesized commercially, and 5’-end labelled with fluorochromes (Syntol, Russia). EUB 338 (5’-GCT GCC TCC CGT AGG AGT-3’) was 5’ labelled with FAM (Ex/Em 488/491 – 575 nm) and used to visualize bacterial eDNA (Amann et al., 1990). STR 405 (5’-TAG CCG TCC CTT TCT GGT-3’) was 5’ labelled with Сy5.5 (Ex/Em: 635/645 – 800 nm) and used to visualize eDNA from oral streptococci (Paster et al., 1998). The Bacto 1080 probe (5’-GCA CTT AAG CCG ACA CCT-3’) was labelled with Acridine (Ex/Em 405/407 – 496 nm) and was used to visualize eDNA derived from the BPP group (Doré et al., 1998). Samples were incubated with 1 mg/ml of lysozyme derived from chicken egg white (47,000 U/mg, Sigma-Aldrich, Germany) in 0.1 M Tris–HCl, 5 mM EDTA (pH 7.2) solution, for 10 min at 37°C, in order to permeabilize cell membranes. For subgingival samples and biofilms formed on PET films, the lysozyme step was omitted to limit probe penetration of cells. These were fixed overnight in 4% paraformaldehyde in phosphate-buffered saline (PBS, 130 mM NaCl, 3 mM KH_2_PO_4_·2H_2_O, 7 mM Na_2_HPO_4_·12H_2_O, pH 7.2) at 4°C and then washed with phosphate-buffered saline and stored at -80°C until hybridization. Samples were dehydrated with a series of ethanol washes containing 50, 80 and 100% (v/v) ethanol, for 3 min each before incubation with oligonucleotides at a concentration of 4 ng/µl each in hybridization buffer (900 mM NaCl, 20 mM Tris–HCl (pH 8), 30% (v/v) formamide and 0.1% (w/v) sodium dodecyl sulphate). Hybridization was conducted in plastic microtubes at 35°C overnight using a Dry Block Thermostat TBD-120 (Biosan SIA, Latvia) and samples were then incubated for 20 min at 37°C in wash buffer containing 20 mM Tris–HCl (pH 8), 64 mM NaCl and 0.1% (w/v) sodium dodecyl sulphate. After washing, samples were analyzed by CLSM as described above.

### Identification of peridontal pathogens

The Vitacell Clinical Laboratory (Ukraine) was used to identify *Actinobacillus actinomycetemcomitans*, *Porphyromonas gingivalis*, *Prevotella intermedia*, *Tannerella forsythus* (*Bacteroides forsythus*), and *Treponema denticola* in subgingival biofilm samples using a Stomatoflor Real Time kit (DNK-Technologia, Russia) (Volinska and Bondarchuk, 2016) and Real-time CFX96 Touch system (BIO-RAD, USA).

### Statistical analyses

Experimental replicates (*n*) were used and quantitative results presented using whisker plots with the median and mean indicated or as bar plots with the mean and standard deviation (SD). One-Way ANOVA using (www.originlab.com) where p < 0.05 was considered statistically significant.

## Results and discussion

### Composition of subgingival-associated biofilms during periodontal disease

**Figure 1:**
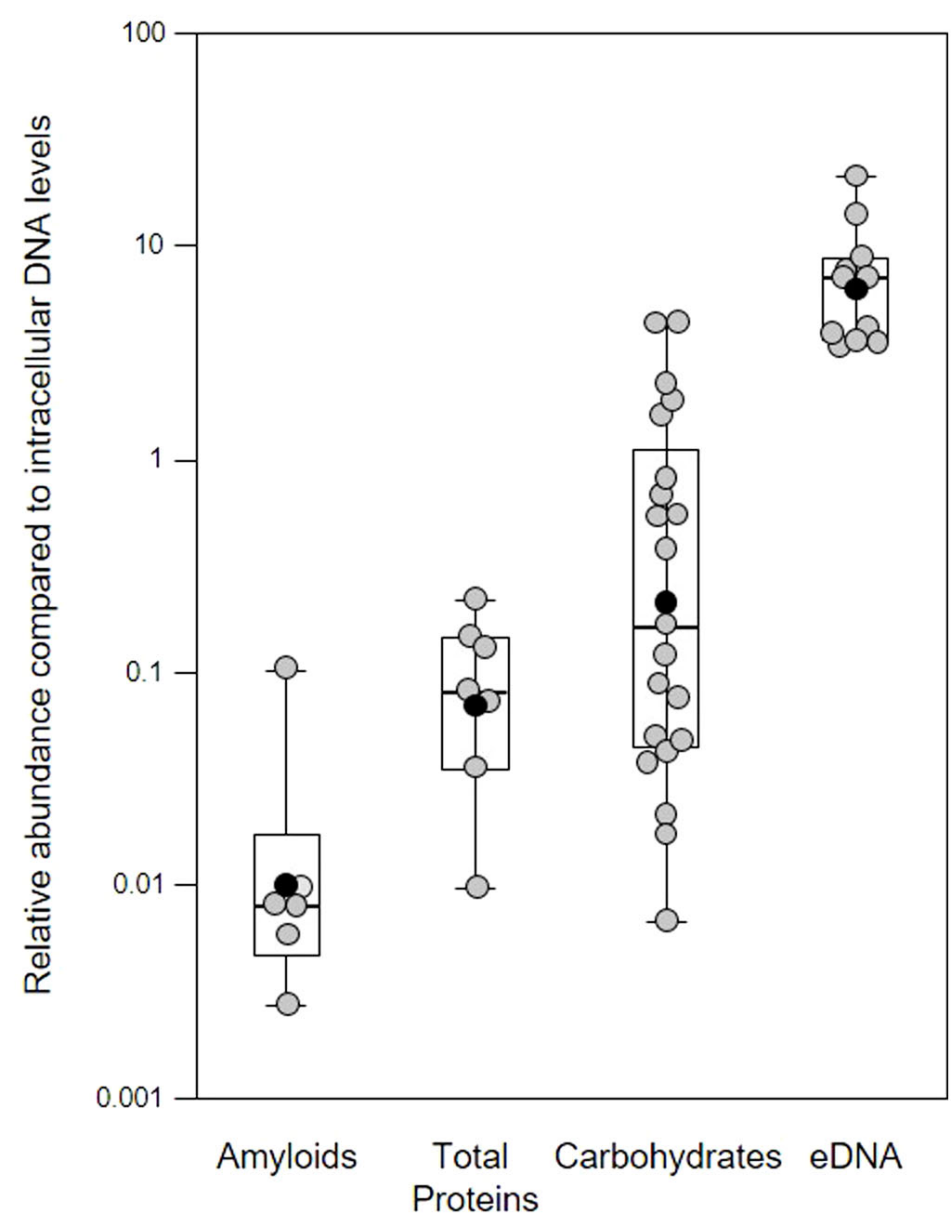
Quantification of extracellular polymeric substances in subgingival biofilms using multiple fluorescent stains. Shown here are the relative abundance of amyloids, total protein, carbohydrates and Candidia cells, and eDNA compared to intracellular DNA (iDNA) levels determined from CLSM images of subgingival biofilms sampled from four patients. Samples were further divided into subsamples to allow staining with multiple dyes. Amyloids stained with AmyGreen, total protein with Thiazine Red R, carbohydrates and Candidia with Calcofluor White, eDNA with Propidium iodide, and iDNA with Ethidium bromide or SYBR Green. Dye mixtures were chosen to avoid overlapping excitation/emission characteristics and subsamples were stained with either AmyGreen, Calcofluor White and Ethidium bromide, Cacofluor White, SYBR Green and Thiazine Red R, or Calcofluor White, Propidium iodide and SYBR Green. Whisker boxplots are shown with the median, mean (black circles) and individual ratios (grey circles). For each material at least three images were quantified from subsamples obtained from patients attending the clinic at different times (pseudo-replicates, *n* = 6 – 21).

Different extracellular polymeric substances (EPS) provide structural integrity to biofilms and a variety of EPS including extracellular DNA (eDNA) contribute to polymicrobial supragingival biofilms (Jakubovics et al., 2021). However, in comparison little is known about the biofilm matrix in the subgingival region during periodontitis. As part of our preliminary investigation, we sampled biofilms from patients with periodontitis with a spoon excavator, stained with different combinations of fluorescent dyes (McLaughlin et al., 2017; Moshynets et al., 2020; Moshynets et al., 2022c), and quantified amyloids, total protein, carbohydrates and Candidia cells, and eDNA relative to intracellular DNA (iDNA) levels (**Figure 1**). This demonstrates the variation seen in biofilm EPS components and the difficulty in establishing normal compositions which may vary within and between patients, and changes resulting from disease progression and possible changes in community composition. While we note variation in total proteins and carbohydrates and Candidia cells in our biofilm samples, we feel the relative abundance of eDNA and amyloids is more interesting, as eDNA is a common matrix component of biofilms encompassing cells within a physical network of fibers, and amyloids interact with eDNA and surfaces to promote attachment (Gallo et al., 2015; Schwartz et al., 2016; Tursi et al., 2017).

In our samples, the mean relative eDNA levels compared to iDNA was 7.75x, suggesting substantial amounts of eDNA matrix surround bacterial cells in these biofilms, and this is supported by CLSM biofilm images (see later figures) where eDNA and iDNA are spatially differentiated. However, some caution is also required in interpreting these results, as normalizing data across biofilm samples using iDNA levels may not always be appropriate (e.g., mature biofilms may have a higher microbial load compared to the eDNA matrix compared to younger biofilms; higher levels of host immune response might have lysed more cells without altering the matrix, etc.).

**Figure 2:**
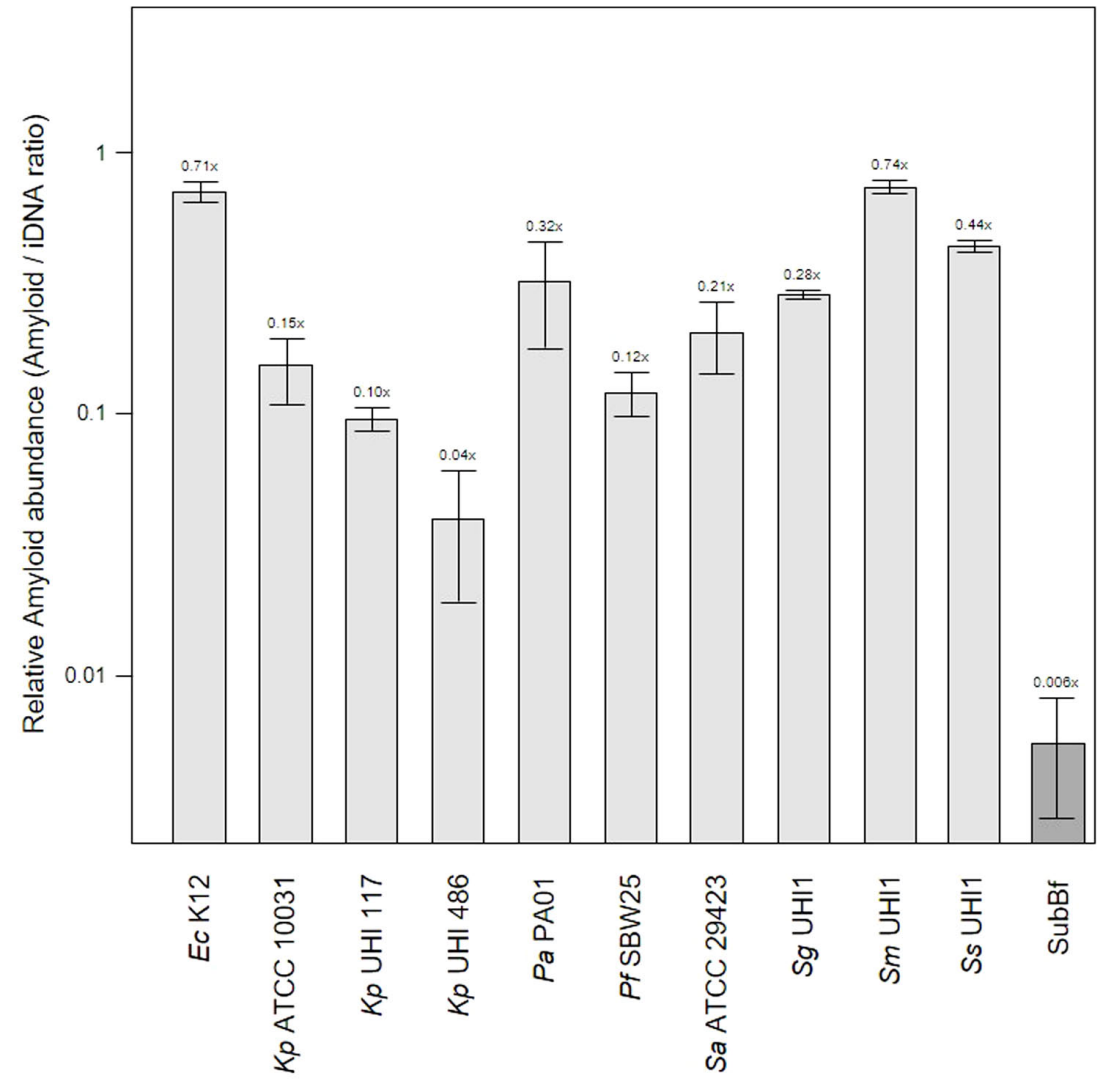
Amyloid levels are high in single-species biofilms compared to subgingival biofilms. Shown here is the relative abundance of amyloids to iDNA determined from CLSM images of three-day old single-species biofilms produced by *Escherichia coli* K12, *Klebsiella pneumoniae* ATCC 10031 and two hospital-acquired *K. pneumoniae* isolates UHI 117 and UHI 486, *Pseudomonas aeruginosa* PA01, *Pseudomonas fluorescens* SBW25, *Staphylococcus aureus* ATCC 29423, *Streptococcus gordonii* UHI1, *Streptococcus sanguinis* UHI1, *Streptococcus mitis* UHI1. The relative amyloid levels from a subgingival multispecies biofilm (SuBf) sampled from a patient also presented in **Figure 1** is shown for comparison. Amyloids were stained with AmyGreen and iDNA with Ethidium bromide and relative abundance calculated from the channel pixel sums as amyloid/iDNA. The relative ratio is also indicated above each bar showing means ± SD (*n* = 3 views from one biofilm sample per strain).

Functional amyloids (i.e., amyloid fibers) are produced by a number of periodontal pathogens including *Streptococcus mutans* and representatives of the red complex of periodontal pathogens (Besingi et al., 2017). One of these, *Porphyromonas gingivalis*, was shown to utilize amyloids for biofilm formation, with at least six variants of amyloid-like fimbrillin (FimA) being produced by this species (Amano et al., 2004; Cueno et al., 2015). Another member of the red complex, *Tannerella forsythia*, also produces the leucine-rich repeat protein BspA, which is potentially amylogenic as predicted by the Waltz algorithm, and is an important adhesin for host receptors such as glycoprotein 340 (Loimaranta et al., 2009). In our samples, the mean relative amyloid level was 0.007x suggesting very low levels of these fibers were present in the biofilms. We investigated this further by determining amyloid levels in single-species *in vitro* biofilm produced by *Klebsiella pneumoniae*, *Pseudomonas aeruginosa*, *Staphylococcus aureus*, and three oral-associated streptococci including *Streptococcus gordonii*, *S. sanguinis* and *S. mitis*, recognized as the early tooth surface colonizers (Rickard et al., 2003), with *Escherichia coli* and an environmental isolate of *Pseudomonas fluorescens* included as additional biofilm reference strains (**Figure 2**).

Amyloid levels relative to iDNA in the single-species biofilms varied from 0.04x for the weak producer *Kp* UHI 486 we have characterized before (Moshynets et al., 2022a) up to 0.71x and 0.74x for amyloid-superproducers *Ec* K12 and *Sm* UHI1. *Sa* ATCC 29423*, Sg* UHI1 and *Ss* UHI, also known to produce considerable amounts of amyloids and are early colonizers of teeth (Ximénez-Fyvie et al., 2000; Larsen and Fiehn, 2017), had similarly high relative levels of amyloids in these single-species biofilms. Interestingly, we note that the two pathogenic *K. pneumoniae* isolates tested here produced lower levels of amyloids compared to the ATCC reference strain that may now be laboratory-adapted.

**Figure 3:**
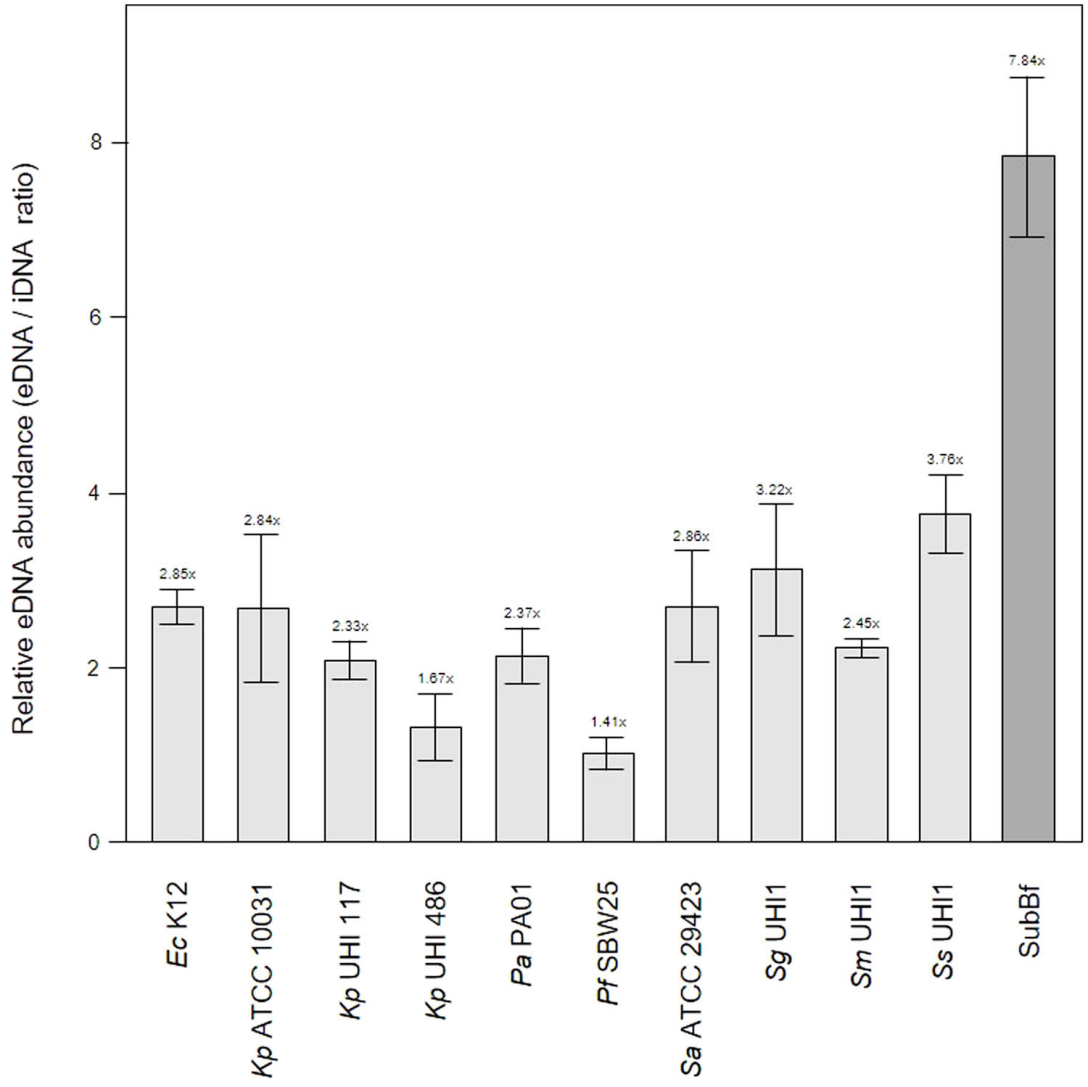
Extracellular DNA (eDNA) is a significant component in single-species biofilms and subgingival biofilms. Shown here is the relative abundance of eDNA to iDNA determined from CLSM images of three-day old single-species biofilms produced by *Escherichia coli* K12, *Klebsiella pneumoniae* ATCC 10031 and two hospital-acquired *K. pneumoniae* isolates UHI 117 and UHI 486, *Pseudomonas aeruginosa* PA01, *Pseudomonas fluorescens* SBW25, *Staphylococcus aureus* ATCC 29423, *Streptococcus gordonii* UHI1, *Streptococcus sanguinis* UHI1, *Streptococcus mitis* UHI1. The relative eDNA levels from a subgingival multispecies biofilm (SuBf) sampled from a patient also presented in **Figure 1** is shown for comparison. eDNA was stained with SYBR green and iDNA with Propidium iodide and relative abundance calculated from the channel pixel sums as eDNA/iDNA. The relative ratio is also indicated above each bar showing means ± SD (*n* = 3 views from one biofilm sample per strain).

The relatively low level of amyloids in our subgingival biofilms might be explained by a low number of amyloid producers or by downregulation of amyloid genesis to avoid or mitigate the immune response, as described for BspA in the *T. forsythia* infection model (Inagaki et al., 2005). Further evidence of an immunogenic effect of amyloid fibers in biofilms has been seen when bound to eDNA (Gallo et al., 2015; Tursi et al., 2017). However, we found lower eDNA levels relative to iDNA in our mature single-species biofilms of 1.41 – 3.76x compared to the subgingival biofilm samples we had sampled earlier with eDNA levels of 7.84x (**Figure 3**). Interestingly, amyloids are known to stabilize eDNA in biofilms and, reciprocally, eDNA promotes amyloid expression in *Enterobacteriaceae* (Schwartz et al., 2016; Taglialegna et al., 2016; Yan et al., 2020). However, this may not occur or be as relevant in subgingival biofilms as we have observed high levels of eDNA and low levels of amyloid. This in turns raises the question of whether all of the eDNA we have identified is of bacterial origin or if some of it is derived from host cells.

One of the leading eDNA producers is *P. aeruginosa* which utilizes eDNA for the initiation and maturation of biofilms (Subendu, 2020; Campoccia et al., 2021; Moshynets et al., 2022b) and is also capable of recruiting host eDNA during maturation (Walker et al., 2005). As our subgingival biofilms contained even more eDNA than that seen in our *Pa* PA01 biofilms or biofilms produced by other reference strains, it is likely that subgingival biofilm development also incorporates host eDNA during development. eDNA is a common component of natural dental plaque formation (Rostami et al., 2017) and is probably also common in the subgingival niche where the most obvious source are neutrophils attracted by an inflammasome during periodontal diseases. A number of studies have described the role of extracellular DNA traps (NETs) produced by neutrophils during periodontitis (Vitkov et al., 2009; Yousefi et al., 2020). However, the contribution of host eDNA derived from NETs to the development and persistence of pathogenic biofilms in periodontitis remains unclear.

### Subgingival biofilms developing during periodontal disease

The importance of local environmental conditions and the econiche for biofilm development and community function has been recognized since the pioneering work in microbial ecology by Winogradsky (Moshynets et al., 2013). However, the modern understanding of biofilm structure, development, and function, has emerged largely from investigations of *in vitro* model systems using a variety of stationary microcosms (Koza et al., 2011; Robertson et al., 2013; Koza et al., 2017) or dynamic flow chambers (Chin et al., 2006; Tolker-Nielsen and Sternberg, 2011). Naturally occurring biofilms develop during colonization of an appropriate surface by multiple species assembling from the local environment and selected by niche physical-chemistry and topography, available resources, competition and predation. This process can be investigated by direct sampling and might reveal important interactions not seen when investigating single-species biofilms using *in vitro* systems, and was first attempted by Cholodny in the 1930s using a glass-slide sampling procedure for soil and subsequently modified by us to use PET films more suitable for modern microscopy techniques (Moshynets et al., 2011; Moshynets et al., 2012). Similar to soil biofilms, the architecture and spatial arrangement of pathogenic biofilms is also an important factor in understanding their persistence and impact on the host. Dental-associated biofilms form on tooth and soft-tissue surfaces following bacterial colonization and can result in complex heterogenic multispecies communities (Rickard et al., 2003). Polytetrafluoroethylene (PTFE, Teflon) carriers have been previously used to sample subgingival bacterial plaque (Wecke et al., 2000).

Dental-associated biofilms can be readily sampled though the procedures used may damage the structure and underlying relationships between host and microbial cells, matrix, and substratum (Wood et al., 2000), and there is little information on pathogenic biofilm structure *in situ* largely because of this issue. The relationship between the biofilm and host cells and substratum is of particular interest since any effective treatment should be developed based on a realistic understanding of the cytological landscape. Although microbial diversity has been reported for subgingival biofilms, limited information is available for spatial structure (Rickard et al., 2003; Ansiliero et al., 2021) and analysis of crevicular exudate from patients with periodontitis has confirmed the importance of neutrophil extracellular traps (NET)-derived eDNA in pathological processes (Vitkov et al., 2009).

**Figure 4:**
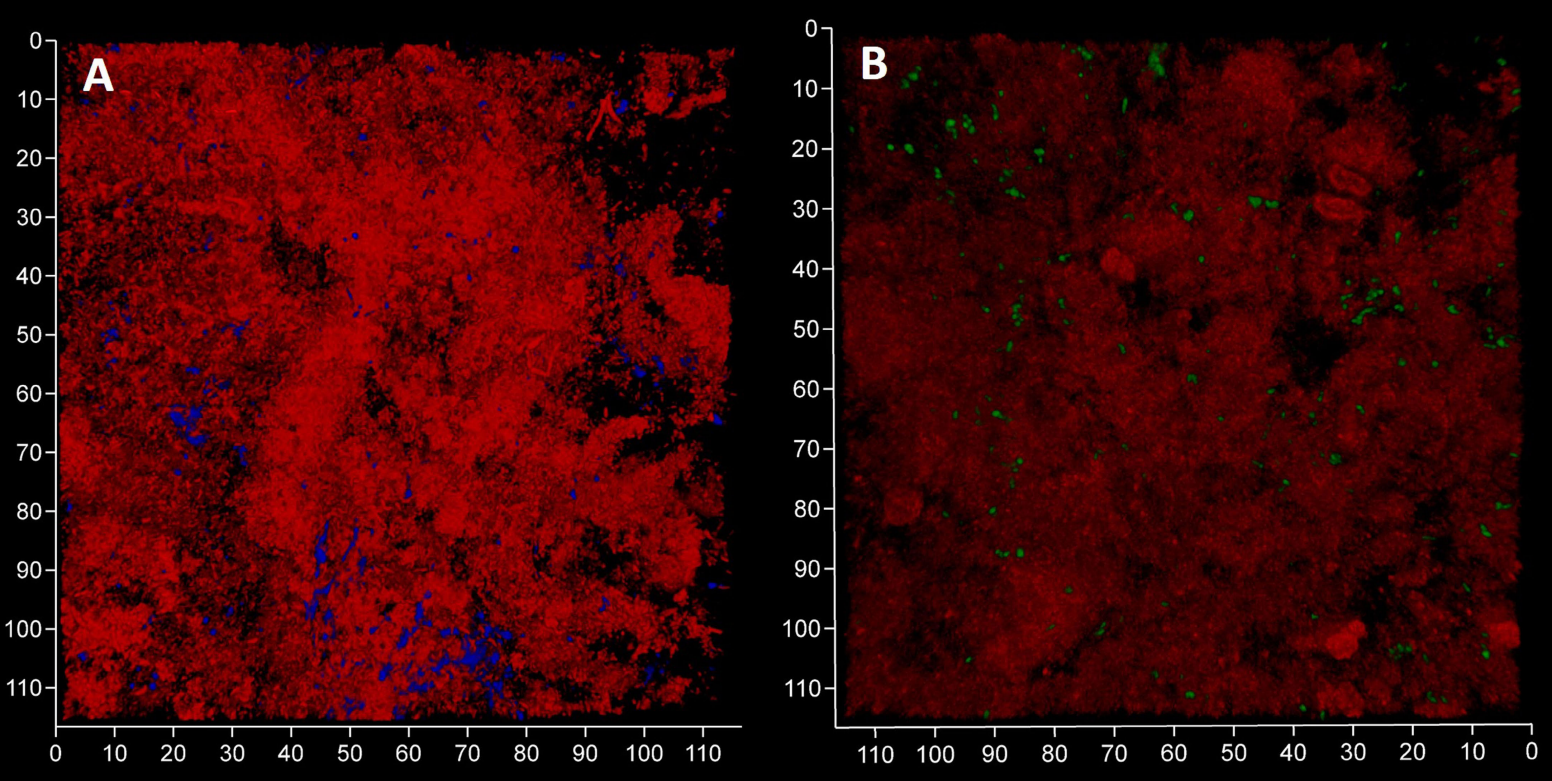
Subgingival biofilms sampled by spoon excavator are dominated by bacterial cells and eDNA. Shown here are representative CLSM images of iDNA from metabolically active and inactive bacterial cells visualized with Ethidium bromide (red channel) and Candida cells with Calcofluor White (blue channel) **(A)** and eDNA visualized with Propidium iodide (red channel) and iDNA in metabolically active cells with SYBR Green (green channel; but note the FRET effect hides the SYBR Green signal when eDNA is also bound by Propidium iodide) **(B)**. The biofilm sample was subdivided in two and stained separately for the images shown here. The axes show 110 µm.

We expected to observe a standard cytological picture of inflammation when sampling periodontal subgingival biofilms from patients using a spoon excavator. However, our CLSM images suggest that these were dominated by bacterial cells and relatively few leukocyte-like cells (**Figure 4**) and suggests that we sampled the top surface of the biofilm rather than the substratum where more leukocytes might be expected to be found. Visualisation of eDNA and iDNA suggested a degree of heterogeneity in the samples with both dense and relatively sparse regions of cells and/or eDNA evident. We also noted the presence of Candidia cells stained with Calcofluor White (**Figure 4A**) and that relatively few metabolically-active bacterial cells were observed in these subgingival samples (**Figure 4B**).

**Figure 5:**
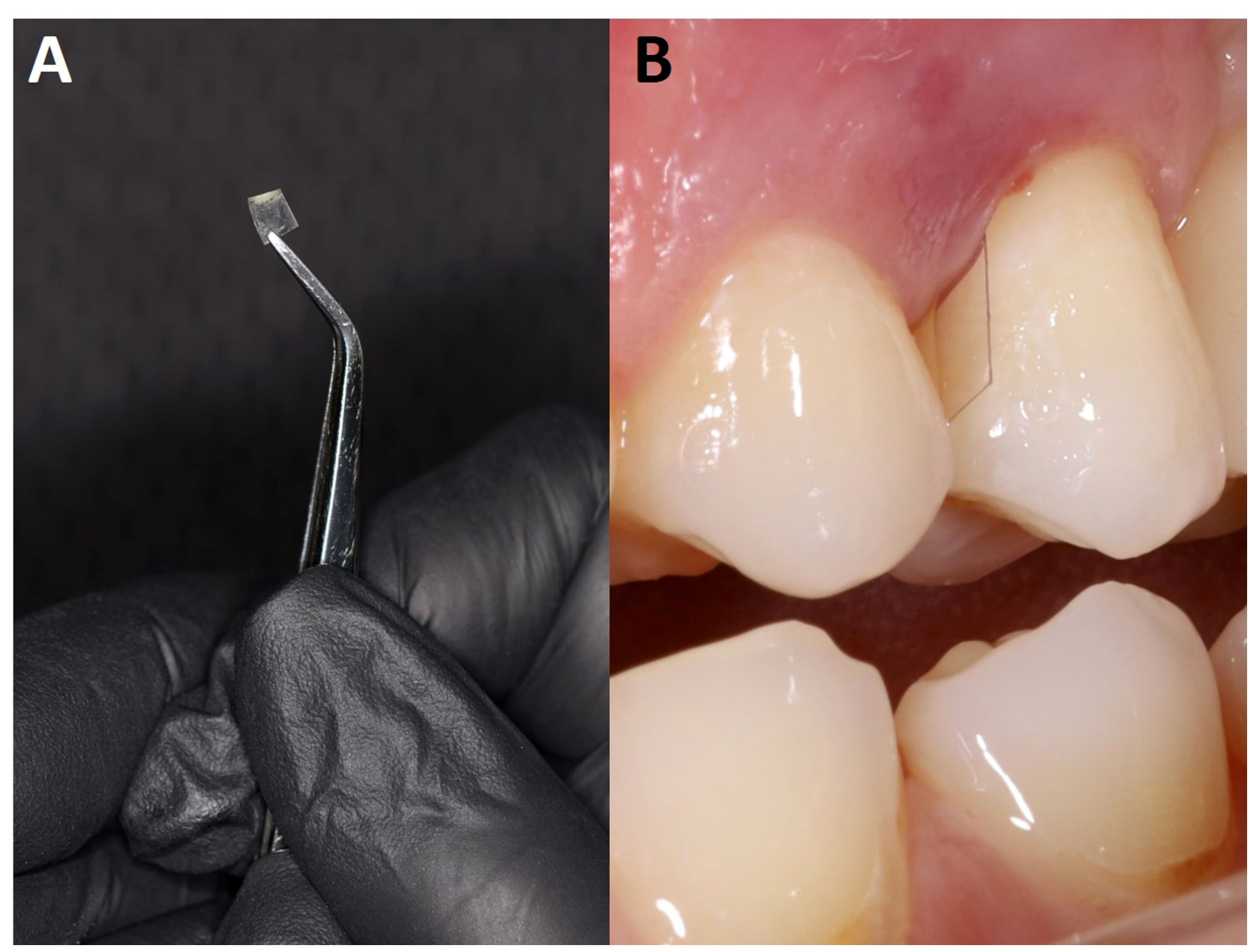
Polyethylene terephthalate (PET) films used to sample subgingival biofilm development. Shown here is a PET film (2 – 4 x 7 mm) **(A)** positioned in the periodontal space next to T24 **(B)**.

**Figure 6:**
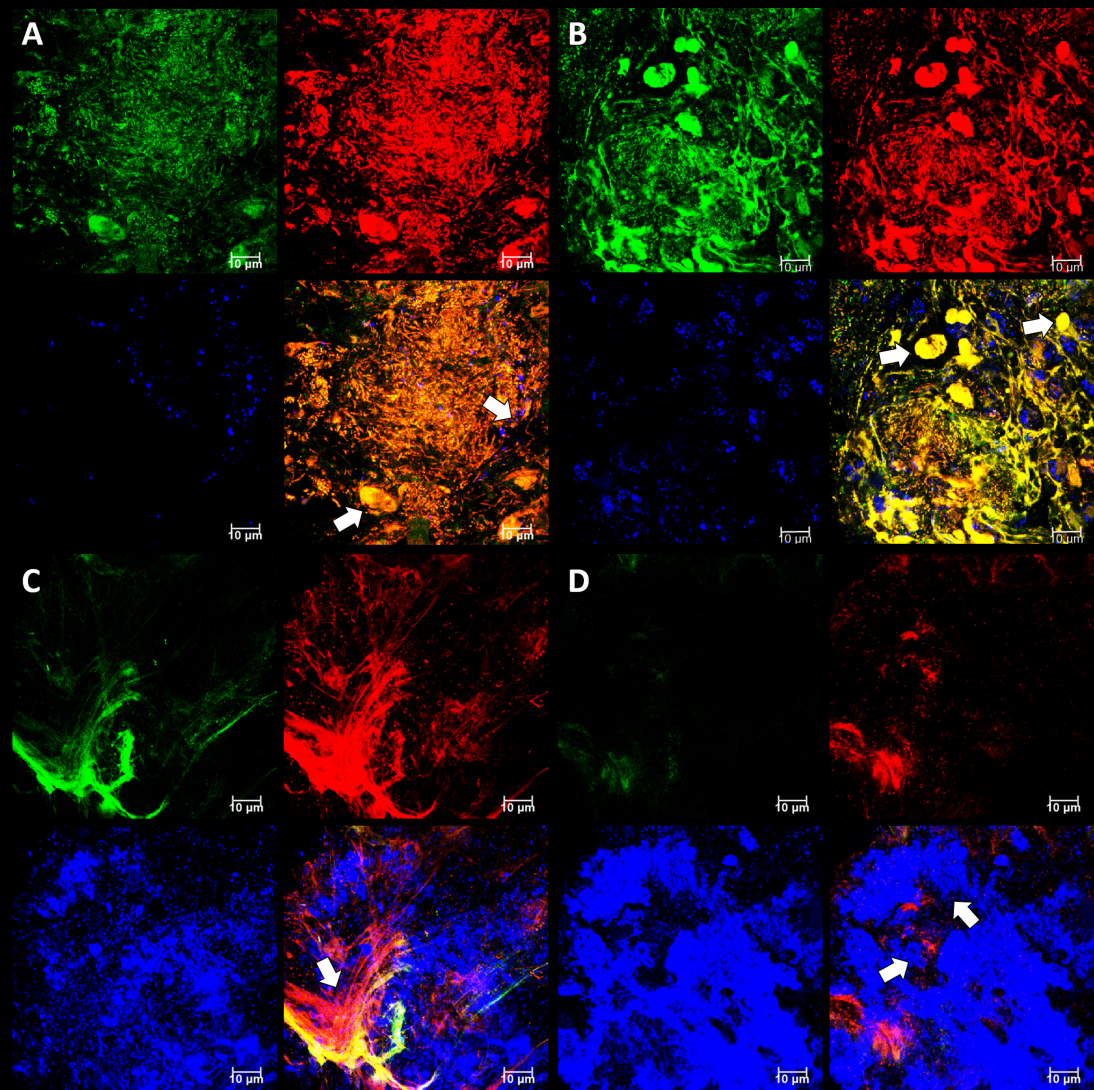
Subgingival micro-landscapes seen developing on PET films during periodontitis. Shown here are representative CLSM images with DNA stained with SYBR Green (green channel), eDNA stained with Propidium iodide (red channel), and Candida cells stained with Calcofluor White (blue channel) of sites with moderate **(A)** and strong inflammation **(B–D)**. The image shown in **(A)** is of a biofilm 12 µm thick with a slice taken 3.3 µm from the plastic surface, and arrows indicate leukocyte and Candida cells. The image shown in **(B)** is of a biofilm 11 µm thick with a slice taken at 7.5 µm and arrows indicate numerous leukocytes. The image shown in **(C)** is of a biofilm 10 µm thick with a slice taken at 1.85 µm and the arrow indicating a fibrin clot. The image shown in **(D)** is from the same position as **(C)**, but the slice is taken at 4 µm and arrows indicate Candida cells. Scale bars show 10 µm.

In order to image undamaged subgingival biofilms, we placed PET films in dental pockets of patients and left them there for five days before recovery (**Figure 5**). This approach is similar to the use of PFTE or enamel fragments to sample dental plaque (Wecke et al., 2000; Wood et al., 2000) but our films are easier to prepare as it does not require a rigid mount and has no biorisks associated with the use of donated material. The PET film will be rapidly coated in a pre-conditioning layer of host and bacterial material, and it also provides a surface for migratory leucocytes which transform into terminal-effector cells such as neutrophils in infection sites. Once colonized the PET film develops a 3D micro-landscape structure representative of host-biofilm interactions in the subgingival niche during periodontitis. As expected, the micro-landscape was dependent upon inflammation intensity and depth of observation (**Figure 6**). More bacteria and a broader morpho-diversity and fewer leukocytes were observed when inflammation was moderate and fewer bacteria and a lot of fibrin-embedded eDNA were observed when the inflammation was strong as was observed earlier (Krautgartner et al., 2010). Candida cells dominated the landscape when the inflammation intensity was high and were generally found at superficial locations (**Figure 6D**).

**Figure 7:**
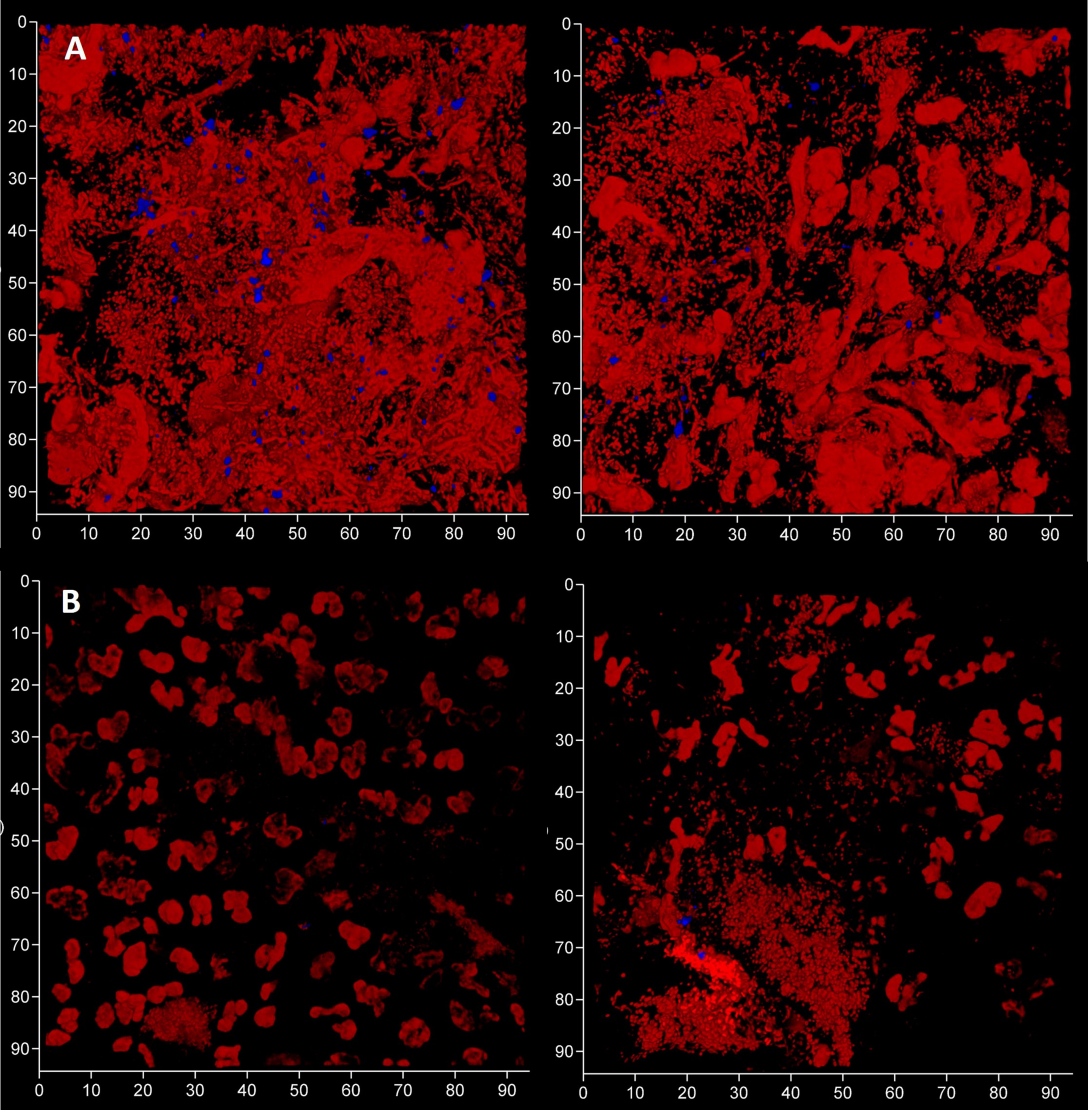
Subgingival micro-landscapes developing on PET films during periodontitis are sensitive to DNAse treatment. Shown here are two sites (left and right) of a subgingival biofilm recovered using a PET film and imaged by CLSM with total DNA stained with Ethidium bromide and Propidium iodide (red channel) and Candida cells with Calcofluor White (blue channel) before **(A)** and after treatment with DNAse **(B)**. The axes show 90 µm.

**Figure 8:**
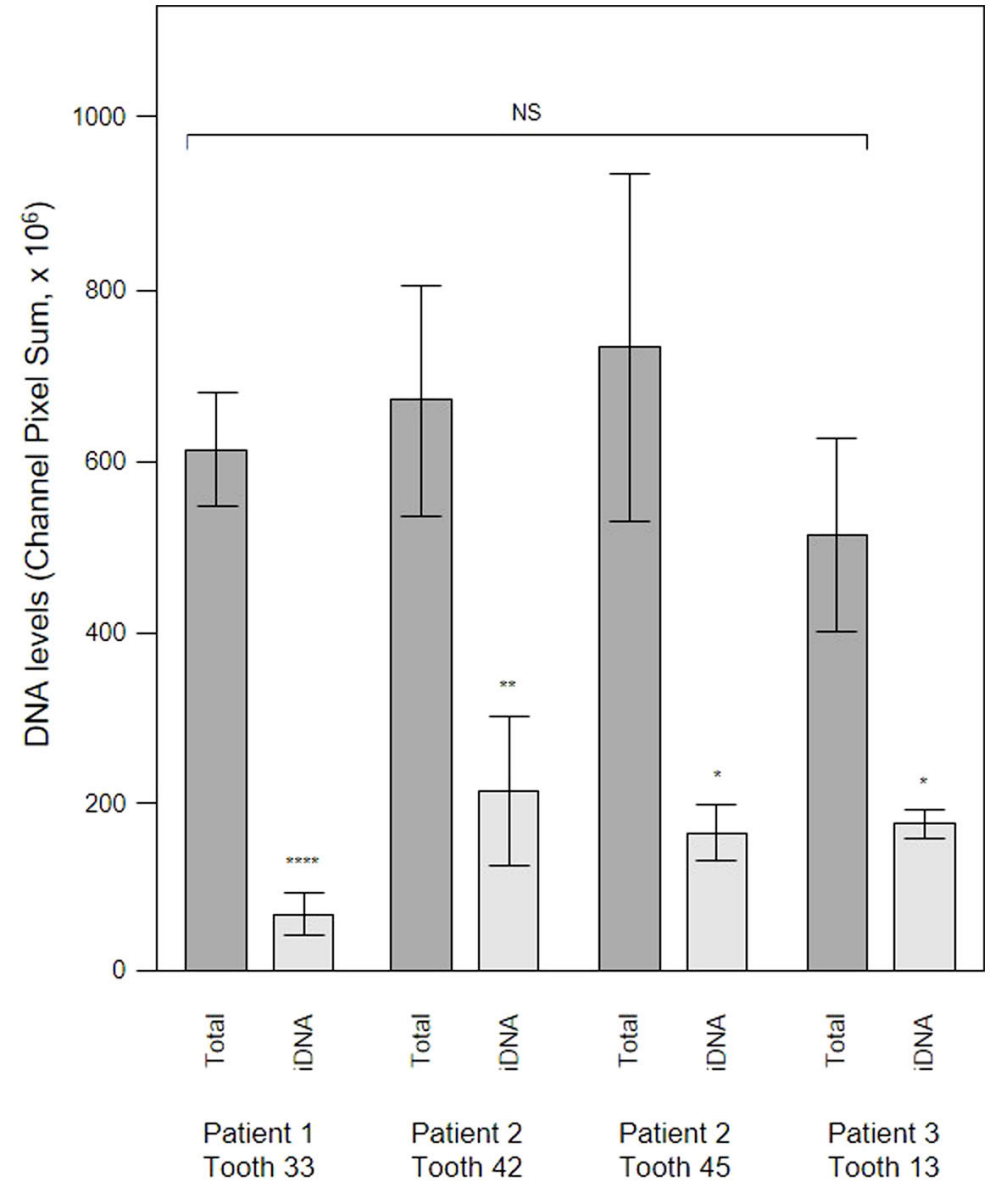
DNAse treatment demonstrates that a significant proportion of the DNA visualized in subgingival biofilms is extracellular DNA (eDNA). Shown here are DNA levels determined from CLSM images of subgingival multispecies biofilms developing in tooth pockets on PET films (Total) which were then treated with DNAse iDNA). DNA removed by DNAse treatment is exposed DNA, i.e., eDNA. DNA levels were calculated from the channel pixel sums. Bars show means ± SD (*n* = 3 views from one biofilm sample per tooth). *p < 0.05, **p < 0.01, and ****p < 0.001; NS, not significantly different at p < 0.05. Patient (P) and tooth (T) numbers are provided in parentheses.

To evaluate the contribution of eDNA to the pathological micro-landscape, PET films were applied to four dental pockets of three patients with initial periodontitis. After five days the films were recovered and cut in half, with one piece treated with DNAse for 30 minutes before staining for total DNA and Candida cells and visualization by CLSM. Representative images from one patient clearly show a substantial reduction of the total DNA signal following DNAse treatment (**Figure 7**) suggesting that most of the signal was exposed eDNA. It is also notable that far fewer Candida cells remained after treatment, suggesting that eDNA helped retain these yeast close to the surface of the tooth. DNA levels were quantified for each of the four PET samples (**Figure 8**) and suggests that eDNA contributes up to ~70% of the DNA visualised using this technique. As the total DNA levels determined for the four micro-landscapes visualised here on PET films were not significantly different, we suggest that they are representative of subgingival multispecies biofilms developing normally in patient’s teeth.

**Figure 9:**
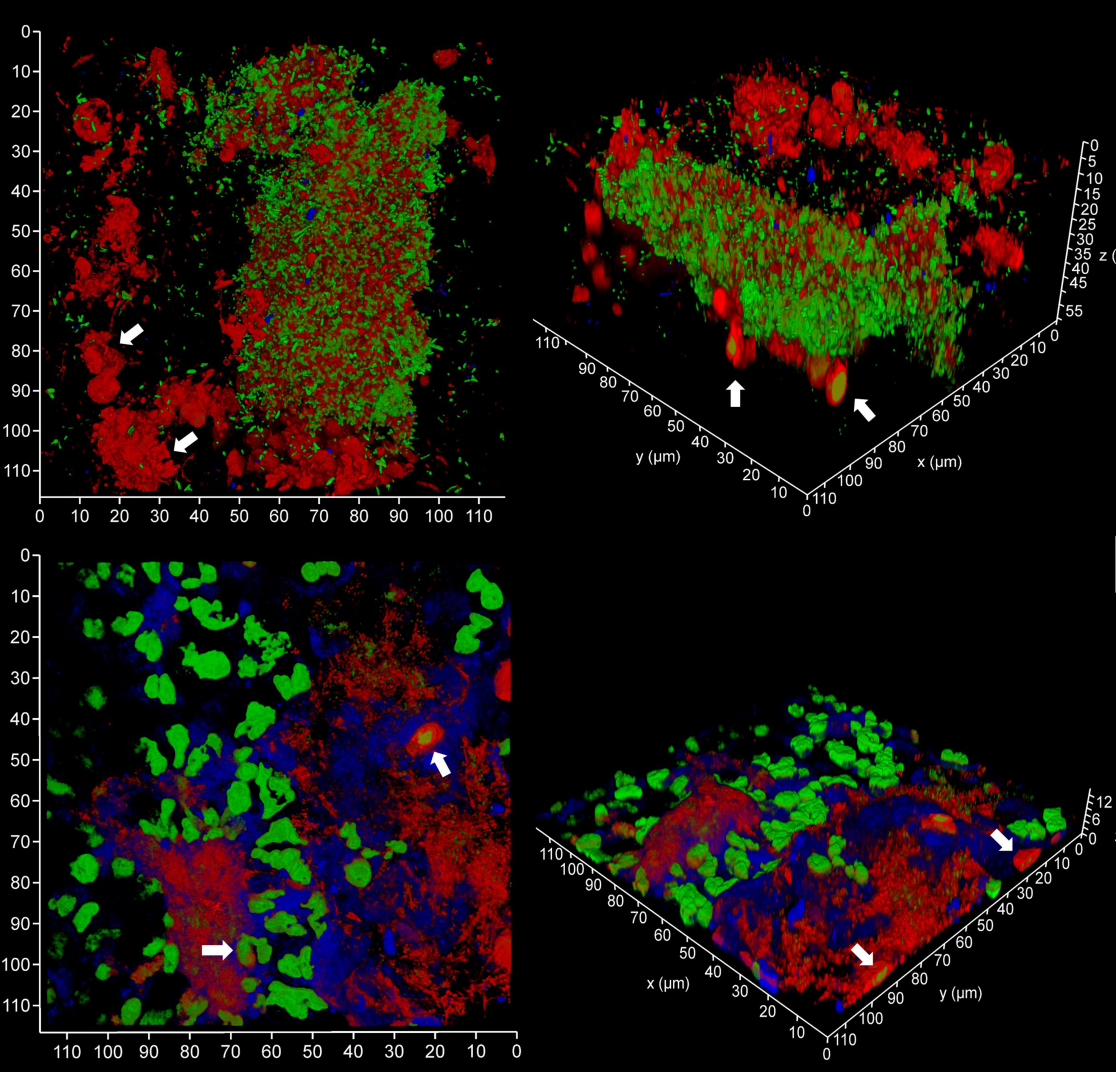
Subgingival micro-landscapes on sampling films during periodontitis. Shown here are two subgingival biofilms recovered using PET film and imaged by CLSM with iDNA stained with SYBR Green (green channel), eDNA with Propidium iodide (top images, red channel) and experimental stain 986 (bottom images, red channel), and Candida cells with Calcofluor White (blue channel). The arrows indicate leukocytes. The *x-y* axes show 110 µm.

A new, experimental, super-heavy DNA-specific stain called “986” with low penetration through intact and damaged membranes specifically developed to visualize eDNA (Moshynets et al., 2022c) was tested using a PET film subgingival micro-landscape (**Figure 9**). eDNA was mainly associated with the biofilm matrix and a superficial surface of leukocytes, and implies that any leukocyte-associated eDNA released at these sites contributes to the bacterial eDNA and the developing biofilm. Previous work using *in vitro* biofilms has shown that neutrophils were able to settle onto *P. aeruginosa* biofilms where they became immobilized, degranulated and transformed their membranes following bacteria direction (Jesaitis et al., 2003). Similar effects where neutrophils activated their functional phagocytosis when attacking *S. aureus* and *S. epidermidis* biofilms have also been observed (Günther et al., 2009; Guenther et al., 2009). Neutrophils sensibilization toward bacterial antigens leads to elevated production of reactive oxygen species, antimicrobial peptides and lactoferrin (Arslan et al., 2009; Stroh et al., 2011; Sol et al., 2013), and when associated with periodontitis results in elevated expression of a Cluster of differentiation (CD) markers panel which leads to a neutrophil phenotype change from parainflammatory in the healthy state to proinflammatory in the diseased patient (Oveisi et al., 2019). This results in phagocytosis, degranulation and neutrophil extracellular traps (NET) activation (NETosis) as a strategy to combat or control pathogenic biofilm development. Such a super-activation of the neutrophils can contribute to the pathogenesis of periodontitis (Kornman, 2008) and might potentially enhance biofilm formation and biofilm-associated phenotypic resistance by periodontal pathogens as seen *in vitro* for *P. aeruginosa* (Yousefi et al., 2020).

The mechanisms of host eDNA elaboration during NET activation has been recently reviewed (Vitkov et al., 2021). The main NETos activators were bacterial outer membrane vesicles heavily loaded with bacterial LPS (Vanaja et al., 2016) and gingipain cysteine proteases produced by periodontal pathogens including *P. gingivalis* (Bryzek et al., 2019) [blood monocytes are also capable of extracellular trap release in response to different stimuli (Granger et al., 2017)]. NETs formed during periodontitis lack bactericidal activity due to the proteolytic activity of the gingipains and do not control pathogenic biofilm development. However, periodontitis-associated NETs are able to stimulate periodontal pathogen growth and possibly biofilm development (Bryzek et al., 2019).

### Host eDNA dominates the microlandscape during periodontal disease

**Figure 10:**
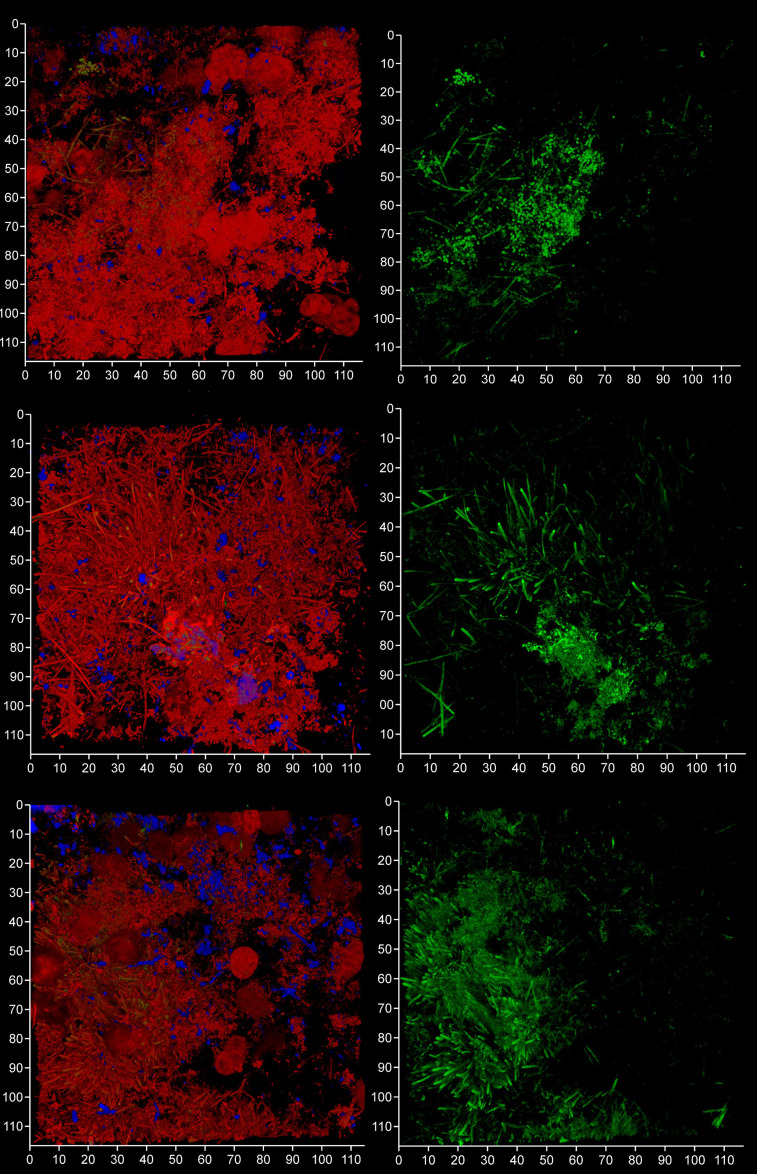
FISH probes can be used to identify bacterial eDNA. Shown here are three subgingival biofilms recovered using PET film and imaged by CLSM after staining eDNA with Propidium iodide (red channel), bacterial eDNA with FISH EUB 338 probe tagged with FITC (green channel), and Candida cells with Calcofluor White (blue channel). The images on the left show all three channels merged while the images on the right show only the green channel. The scale bars show 110 µm.

**Figure 11:**
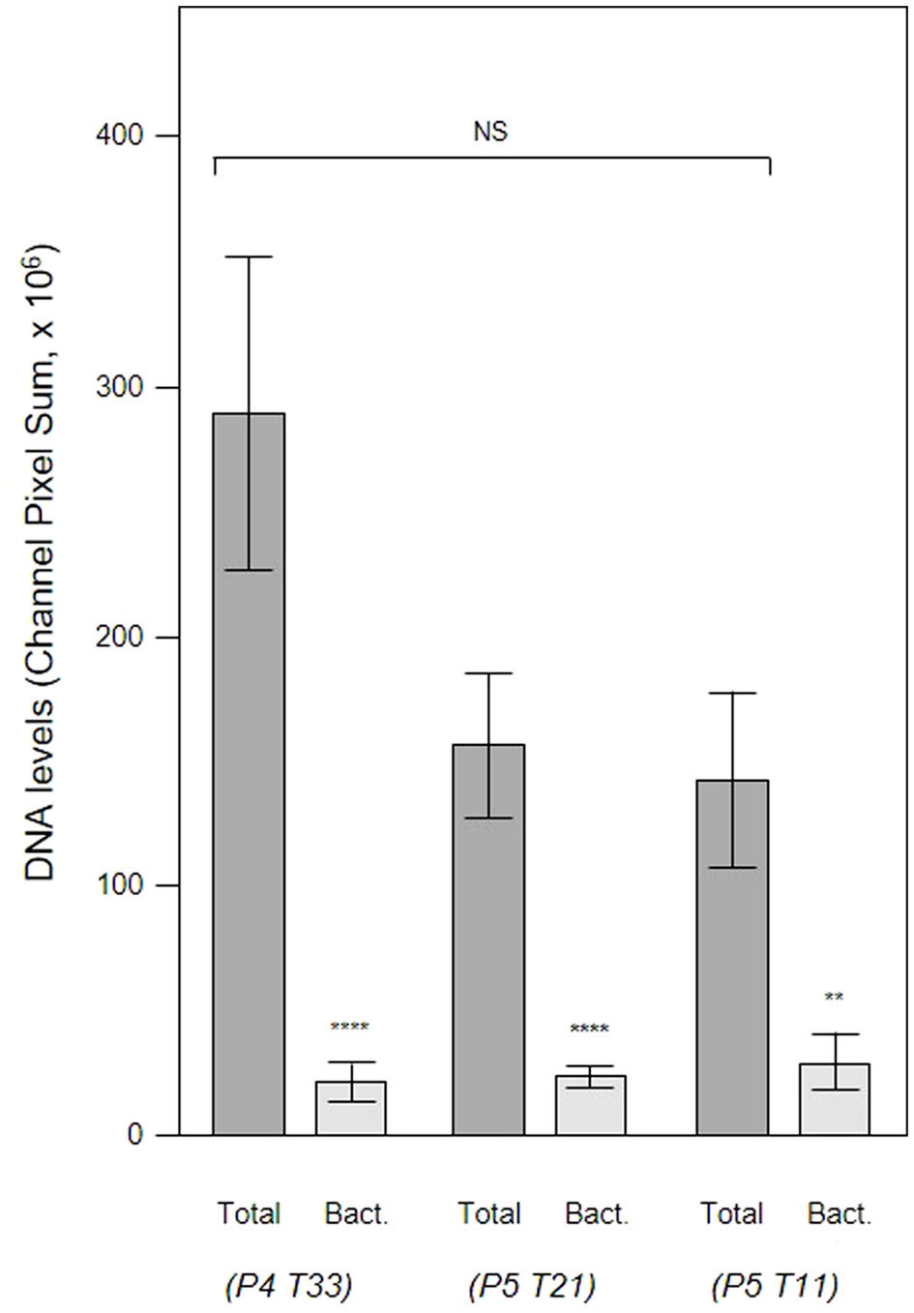
Total eDNA and bacterial eDNA levels in subgingival biofilms. Shown here are the total eDNA and bacterial eDNA (Bact.) levels determined from CLSM images of three subgingival biofilms recovered using PET films stained with propidium iodide and EUB 338. Bars show means ± SD (*n* = 7 views from one biofilm sample per tooth). **p < 0.01 and ****p < 0.001; NS, not significantly different at p < 0.05. Patient (P) and tooth (T) numbers are provided in parentheses.

In order to evaluate the contribution of host and bacteria-derived eDNA, we undertook fluorescent *in situ* hybridization (FISH) of PET films using the EUB 338 bacteria-specific probe (**Figure 10**). Analysis of three samples of subgingival micro-landscapes from two patients shows that bacterial eDNA may contribute 8 – 21% to the total eDNA with the majority derived from host cells activated following periodontitis (**Figure 11**). It is therefore possible that the release of host DNA is responsible for the consistent persistence and pathology chronization.

### eDNA from the *Bacteroides–Porphyromonas–Prevotella* group might be a major bacterial eDNA source in the microlandscape during periodontal disease

**Figure 12:**
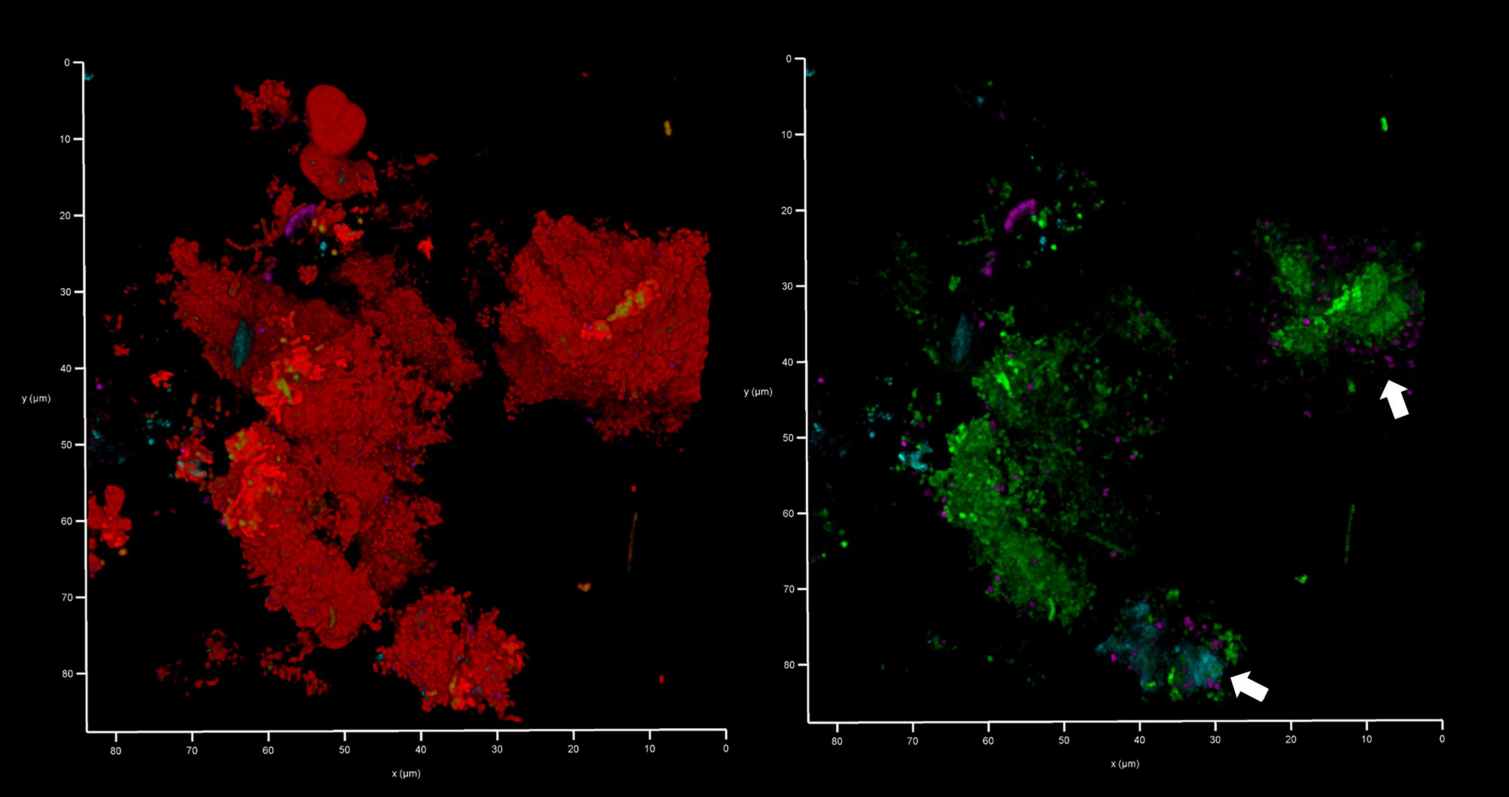
*Bacteroides–Porphyromonas–Prevotella* and oral streptococci group eDNA. Shown here is a subgingival biofilm recovered using a PET film and imaged by CLSM after staining eDNA with Propidium iodide (red channel), bacterial eDNA with EUB 338 probe tagged with FITC (green channel), *Bacteroides–Porphyromonas–Prevotella* group with Bacto 1080 probe tagged with modified Acridine (blue channel), and the oral streptococci group with STR 405 probe with Cy5.5 (purple channel). The left image shows all four channels merged while the right image shows the green, blue and purple channels merged. Arrows point at a *Bacteroides–Porphyromonas–Prevotella* and streptococcal microcolonies. The axes show 80 µm.

**Figure 13:**
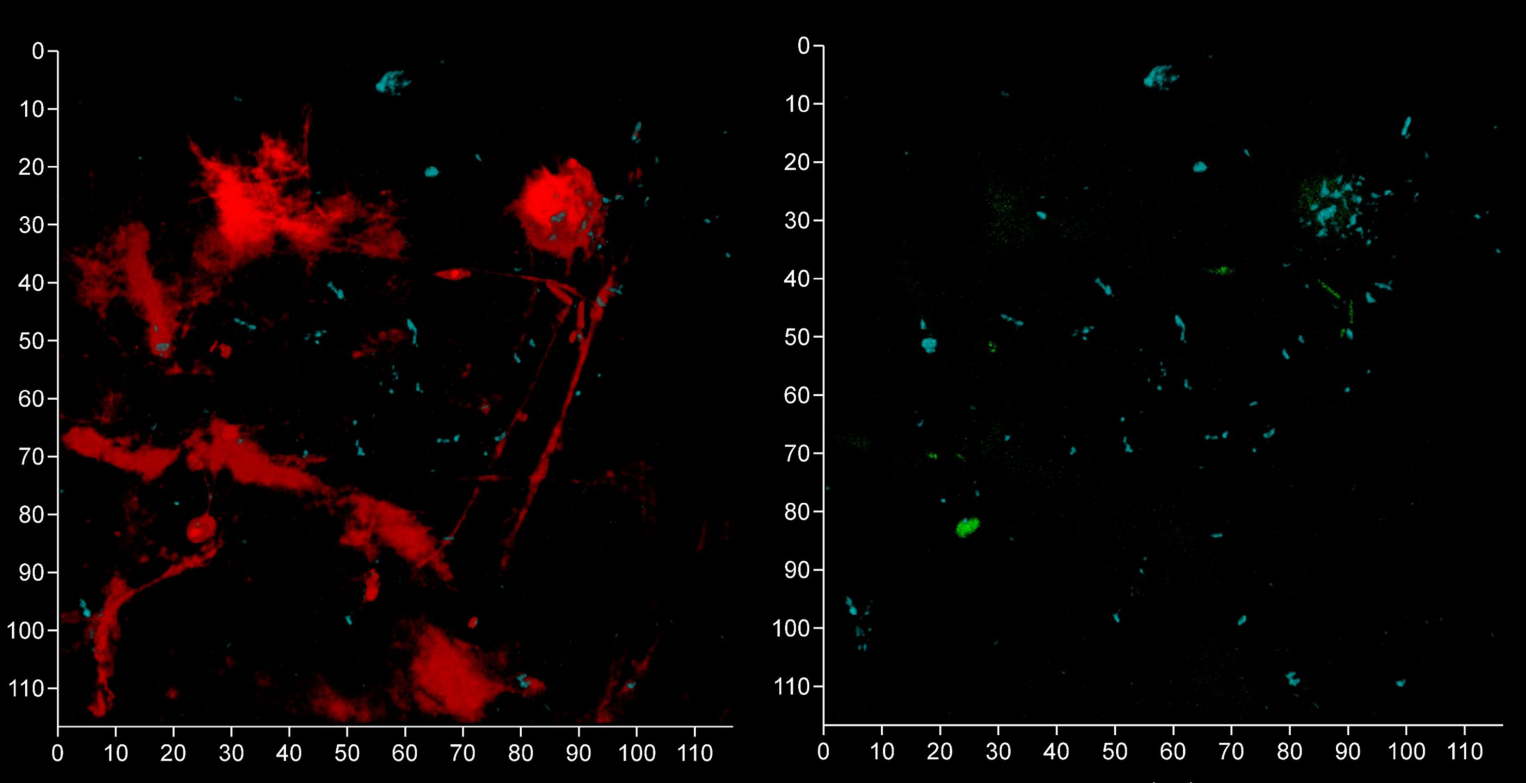
Oral streptococci group bacteria are less common near a fibrin clot. Shown here is a subgingival biofilm near a fibrin clot recovered using a PET film and imaged by CLSM after staining eDNA with Propidium iodide (red channel), bacterial eDNA with EUB 338 probe tagged with FITC (green channel), *Bacteroides–Porphyromonas–Prevotella* group with Bacto 1080 probe tagged with modified Acridine (blue channel), and the oral streptococci group with STR 405 probe with Cy5.5 (purple channel). The left image shows all four channels merged while the right image shows the green, blue and purple channels merged. The axis show 110 μm.

**Figure 14:**
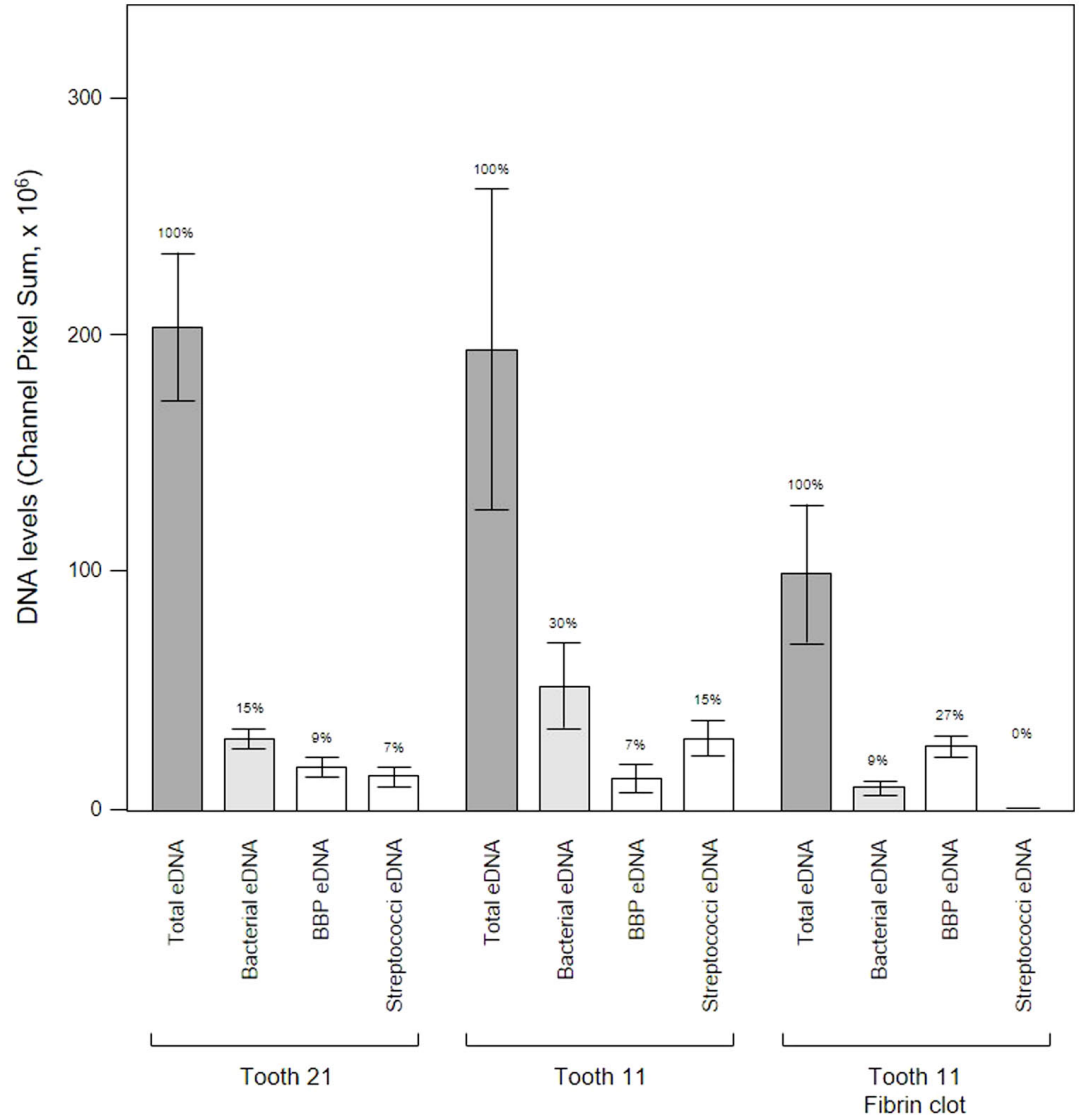
Bacterial eDNA origins differ between teeth and proximity to a fibrin clot. Shown here are the total, bacterial, *Bacteroides–Porphyromonas–Prevotella* group (BPP) and oral streptococci group eDNA levels determined from CLSM images of three subgingival biofilms recovered using PET films stained with Propidium iodide and FISH probes. Bars show means ± SD (*n* = 5 views from one biofilm sample per tooth) and percentage of total eDNA for each site is shown above the error bars.

We analyzed a randomly-chosen clinical case of periodontitis (patient 5) to determine the origin of bacterial eDNA involved in subgingival biofilm formation by real time PCR and CLSM. The PCR analysis confirmed the presence of pathologically significant periodontal pathogens including *Actinobacillus actinomycetemcomitans*, *Porphyromonas gingivalis*, *Tannerella forsythensis, Treponema denticola* and *Candida albicans*. FISH analysis of PET films using the Bacto 1080 probe for the *Bacteroides–Porphyromonas–Prevotella* (BPP) group and the STR 405 probe for the oral streptococci group suggest that these might not be dominant in the subgingival micro-landscape, and as before host eDNA was more abundant than bacterial eDNA (**Figure 12**). We noticed a difference in the subgingival micro-landscape close to a fibrin clot which are commonly found during periodontitis (**Figure 13**). No streptococci were present near the clot although an increased number of anaerobic bacteria were found instead. This might be explained by the antibacterial effects of host eDNA and associated proteins which contribute to the clot when the fibrinogen polymerized. Representatives of the BPP group can neutralize the antibacterial activity of NET-associated host defense proteins using gingipain cysteine proteases (Bryzek et al., 2019) which cannot be done by other colonizers or by streptococci in particular. Analysis of the probe signals allowed us to compare the contribution of eDNA derived from both the BPP group and the oral streptococci group with the total bacterial eDNA which suggest that the anaerobes and streptococci may be the main biofilm-producers in the subgingival niche during periodontitis (**Figure 14**).

## Conclusion

Microbiologically induced inflammation processes underlying periodontitis take place in the subgingival niche. However, most investigations of periodontitis including dental calculus analysis have focused on the supragingival region and there is little information on the subgingival niche and the structure of the micro-landscapes during periodontitis. In this work, we undertook a comparative analysis of the EPS composition of subgingival-associated biofilms and microcosm-grown *in vitro* single-species biofilms including three isolates of oral streptococci. Amyloid genesis was found to be downregulated in the subgingival biofilms and eDNA revealed to be the main biofilm matrix component. Subsequent comparative analysis confirmed that the amount of eDNA in subgingival biofilms was higher than the bacterial biofilms normally generated including the leading eDNA producer *P. aeruginosa* PA01. FISH analysis of subgingival micro-landscapes sampled during periodontal disease revealed that host eDNA was dominant with only 8 – 21% of the total produced by periodontal pathogens. The main source of the host eDNA appears to be immunocompetent cells activated following periodontitis and is therefore a major factor of persistence and pathogenesis while eDNA from the *Bacteroides–Porphyromonas–Prevotella* and oral streptococci groups suggest that these may be the most important biofilm-formers during periodontal disease.

## Data availability statement

The raw data supporting the conclusions of this article will be made available by the authors, without undue reservation.

## Ethics statement

The studies involving humans were approved by the Ethics Committee of from the Shupyk National Healthcare University of Ukraine. The studies were conducted in accordance with the local legislation and institutional requirements. The participants provided their written informed consent to participate in this study.

## Author contributions

MSK: Conceptualization, Data curation, Formal analysis, Funding acquisition, Investigation, Writing – original draft. OM: Conceptualization, Data curation, Formal analysis, Funding acquisition, Methodology, Project administration, Resources, Supervision, Validation, Writing – original draft, Visualization, Writing –review & editing. SK: Investigation, Writing – original draft. IM: Investigation, Writing – original draft. KR: Investigation, Writing – original draft. VP: Investigation, Writing – original draft. OI: Investigation, Writing – original draft. OK: Investigation, Writing – original draft. IK: Investigation, Writing – original draft. GP: Visualization, Writing – review & editing, Writing – original draft. AJS: Visualization, Writing – review & editing, Writing – original draft.
